# Extracellular Vesicles and Exosomes in the Control of the Musculoskeletal Health

**DOI:** 10.1007/s11914-024-00866-2

**Published:** 2024-03-01

**Authors:** Fabio Penna, Lorena Garcia-Castillo, Paola Costelli

**Affiliations:** Department of Clinical and Biological Sciences, University of Turin, Corso Raffaello 30, 10125 Turin, Italy

**Keywords:** Muscle-bone cross-talk, Sarcopenia, Bone loss, MicroRNAs, Therapeutic tools

## Abstract

**Purpose of Review:**

The present review will highlight recent reports supporting the relevance of extracellular vesicles to the musculoskeletal system in health and disease.

**Recent Findings:**

Preserving the health of the musculoskeletal system is important to maintain a good quality of life, and the bone-muscle crosstalk is crucial in this regard. This latter is largely mediated by extracellular vesicles released by the different cell populations residing in muscle and bone, which deliver cargoes, microRNAs, and proteins being the most relevant ones, to target cells. Extracellular vesicles could be exploited as therapeutic tools, in view of their resistance to destruction in the biological fluid and of the possibility to be functionalized according to the need.

**Summary:**

Extracellular vesicles are recognized as crucial players in the bone-muscle cross-talk. Additional studies however are required to refine their use as biomarkers of early alterations of the musculoskeletal system, and as potential therapeutic tools.

## Introduction

Extracellular vesicles (EVs) mediate the intercellular exchange of classical soluble and insoluble signaling factors, as well as of structural proteins, nucleic acids, and lipids. When released from the cell of origin, EVs travel through the interstitial space to reach the circulation. They can be uptaken by distant tissues, where they convey information able to affect the target cell function and/or metabolism and that could ultimately result in the production of other EVs that could go back to the initial source, in a sort of feed-back mechanism. On the basis of their origin, size, physical properties, and functions, different types of EVs have been described, among which exosomes and microvesicles (MVs). Exosomes (50–150 nm) form within the intraluminal vesicles that are generated in the multivesicular bodies as part of the late endosomes. Multivesicular bodies can either fuse with lysosomes for degradation or travel back to and fuse with the plasma membrane. Thus, molecules can be recycled and released into the extracellular space by means of exosomes. Sorting of the cargo during the internal budding of the membrane that leads to intraluminal vescicle formation is an essential step in exosome biogenesis [1]. By contrast, MV (50–1000 nm) generation starts from the formation of outward buds in specific sites of the plasma membrane followed by fission and subsequent release of the vesicle into the extracellular space. Lipid raft domains seem to be abundant in MVs, and MV formation can be impaired by cholesterol depletion. In addition to rearrangements in the plasma membrane composition, proteins responsible for cell shape maintenance may be involved in MV biogenesis [1].

EVs can be secreted by many cell types, including tumor cells. EV content is highly heterogeneous, consisting, in variable proportions, of proteins, DNA, microRNAs (miRs), mRNAs, lipids, surface molecules, etc. Importantly, EVs might represent a vehicle in which nucleic acids can be preserved and analyzed in biological fluids, as well as delivered to their target cells without being degraded in the extracellular space [1]. Due to these features, EVs have been proposed to work as biomarkers [2] and to be exploited as suitable therapeutic tools [3].

Several lines of evidence showed that EVs play a crucial role in the regulation of tissue homeostasis in health and in diseases such as cancer, diabetes, obesity, musculoskeletal, and cardiovascular disorders. The present review will focus on the relevance of EVs to the musculoskeletal system.

## Musculoskeletal Health and Disease

The musculoskeletal homeostasis is maintained due to the biomechanical and humoral interconnection occurring between bone and muscle (bone-muscle cross-talk). Indeed, a parallel growth of muscle and long bones occurs in childhood, reaching a size that is generally maintained and adapted to fit the metabolic and mechanical needs in healthy adults. During aging, bone frailty such as that occurring in osteoporosis is frequently associated with muscle disuse, resulting in sarcopenia. Such a reciprocal regulation also reflects a metabolic cross-talk, in which amino acids, humoral factors, and hormones contribute to maintain the physiological rates of muscle and bone protein turnover, a homeostasis which is disrupted during aging and pathological states. Particularly relevant to the muscle-bone cross-talk are molecules released by both tissues, namely myokines and osteokines, which interfere with tissue metabolism and function, both locally and systemically. As an example, on the muscle side, several released factors, such as insulin-like growth factor (IGF)-1, transforming growth factor (TGF)-β, myostatin, irisin, and matrix metalloproteases, modulate bone deposition and resorption. On the bone side, osteokines include osteocalcin and sclerostin, which have been shown to impinge on the skeletal muscle mass [4, 5]. Another factor that seems relevant to the muscle-bone cross-talk is the molecular clock that regulates the circadian rythms. In this regard, there is a single study reporting that muscle circadian rythms contribute to skeletal health. More specifically, the study was performed on mice in which the clock master regulator Bmal1 was specifically deleted in the skeletal muscle. The lack of Bmal1 resulted in reduced muscle force and increased muscle fibrosis and oxidative stress. However, despite the genetic defect was limited to the skeletal muscle, cartilage and bone were affected as well, with reduced collagen at the joint level and increased bone calcification, respectively [6].

Musculoskeletal tissues mainly originate from mesenchymal stem cells (MSCs), which are able to differentiate into myocytes, osteocytes, chondrocytes, fibroblasts, or adipocytes, depending on the microenvironment. In this regard, modulations of MSC abundance and function may affect the musculoskeletal homeostasis. As an example, MSCs isolated from aged volunteers display reduced proliferative capacity, altered morphology, and increased protein aggregates in comparison to MSCs obtained from young individuals [7]. These features significantly contribute to the alterations in bone mineral density and in skeletal remodeling occurring during aging. On the other side, altered MSC properties could contribute as well to the aging-associated muscle phenotype, by impairing the regenerative capacity of satellite cells [8].

Musculoskeletal health is pivotal to maintain physical performance, overall well-being, and the ability to perform normal daily life activities. Along this line, its preservation crucially affects both independence and quality of life, the more so in elderly people. Consistently, musculoskeletal disorders such as osteoporosis, osteoarthritis, spinal degeneration, low back pain, muscle injury, and sarcopenia are among the most relevant contributors to disability worldwide [9]. The occurrence of these diseases, which is associated with chronic pain and loss of function, frailty, reduced quality of life, and increased risk of falls and fractures, is expected to steadily increase, in particular when the growing number of aged and obese people is considered. Indeed, musculoskeletal disorders are frequent comorbidities in patients affected by other non-communicable diseases, conversely representing a risk factor for the development of multimorbidity, favoring, as an example, the onset of cardiovascular and psychological illnesses [10]. Last, but not the least, the increasing prevalence of musculoskeletal diseases is a considerable economic burden for the social security system.

At present, the available therapy for musculoskeletal diseases is only symptomatic, mainly based on the assumption of anti-inflammatory drugs, surgery being the last option for patients in end-stage disease. The tools available to pick up early alterations of the physiological homeostasis of the musculoskeletal system and then to treat the underlying diseases are still poorly defined. Just as an example, reduced bone mineral density and sarcopenia are significantly correlated with increased risk of falls and fractures [11], and for these reasons should be monitored in the aging population. Several studies addressed the identification of strategies aimed at maintaining the musculoskeletal health as long as possible. In this regard, a lifestyle including physical activity and good dietary habits seems to be crucial [12]. Consistently, evidences have been provided that elderly people regularly performing physical activity show a reduced incidence of falls and fractures [13]. From the nutritional point of view, particularly relevant in this regard is protein, calcium, and vitamin D intake [12].

In addition to the adoption of a healthy lifestyle, several lines of research have been developed to define suitable pharmacological approaches directed to people unable, for any reason, to cope with low-to-moderate physical activity, or to lower the exercise dose required. A lot of interest in this regard was dedicated to drugs able to act as exercise-mimicking agents: by now several types of molecules have been released to the market, even if most of them still are confined to the pre-clinical phase. The number of biological processes covered by these drugs is considerably large, ranging from molecules able to interfere with energy metabolism (AMPK or PPARδ agonists), or muscle and bone trophism (vitamins C and E), or osteogenesis (irisin), to specific molecular targets acting on redox homeostasis, mitochondrial function, inflammation, or myogenesis (Nrf2 and PGC-1α modulators) [4].

In the last decade, however, the possibility to use EVs, functionalized or directly derived from specific cell populations, to preserve, prolong, or restore a healthy musculoskeletal system, has been gaining a growing interest.

## EVs in the Regulation of Muscle and Bone Homeostasis

**Fig. 1:**
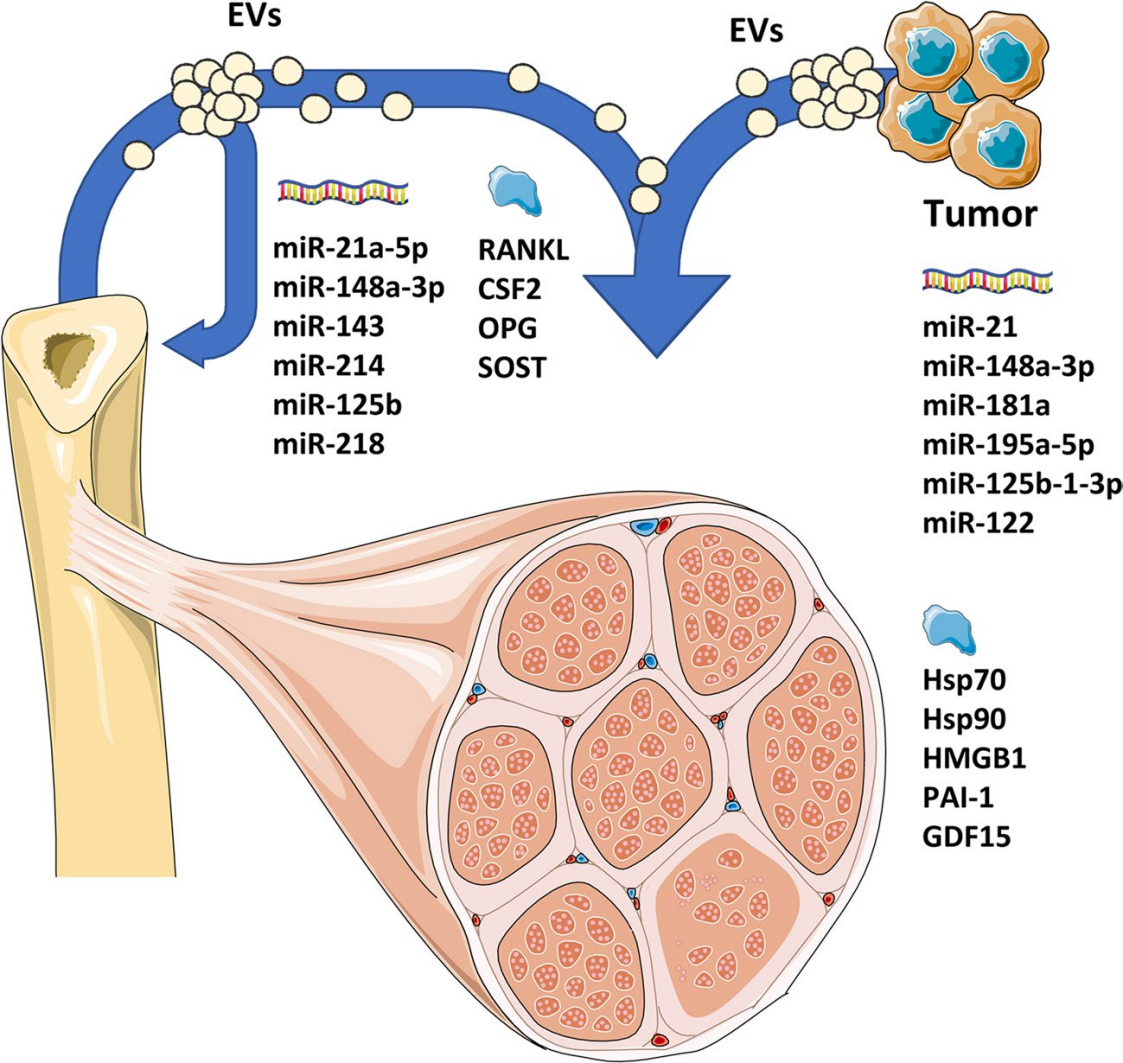
Bone and cancer cells secrete extracellular vesicles, delivering paracrine and endocrine signals. Among other molecules, EV cargo is rich in proteins and microRNAs; a non-exhaustive list of the most relevant ones in determining musculoskeletal disorders is reported in the cartoon. Image created with PowerPoint and Servier Medical Art. EVs, extracellular vesicles; OPG, osteoprotegerin; SOST, sclerostin; HSP, heat shock protein; HMGB1, high mobility group box 1; PAI-1, plasminogen activator inhibitor-1; GDF15, growth and differentiation factor 15

EVs exert their biological function of cell-to-cell communication by transporting and delivering molecules from one anatomical compartment/cell population to the others. For this reason, the identification of the cargoes most relevant to the homeostasis of specific tissues has been widely investigated, showing that non-coding RNAs (microRNAs, in particular) and proteins are mainly involved (Fig. 1).

### MicroRNAs

#### Bone

EVs released by mature osteoblasts have been shown to exert an autocrine inhibition of osteoblast differentiation. Such EVs contain miR-21a-5p, miR-148a-3p, and miR-143. This latter is able to reduce the expression of the transcription factors Runt-related transcription factor 2 (Runx2) and Sp7/Osterix, increasing the expression of the receptor activator of nuclear factor kappa B (RANK) ligand RANKL, a crucial actor in osteoclastogenesis. Such an effect appears to depend on the ability of miR-143 to target the core-binding factor β (Cbfb), which regulates Runx2 transcriptional activity [14]. As for miR-21, its deletion in osteocytes exerts divergent effects in males and females, resulting in increased osteocyte death and inhibition of bone turnover in the latter, while inducing an opposite phenotype in the former. However, such a sex-specific behavior cannot be detected in terms of bone mechanical properties, which are improved both in males and females [15]. Consistently with the occurrence of a bone-muscle cross-talk, miR21 deletion in osteocytes also affects the skeletal muscle. In this regard, increased muscle mass occurs in female mice only, while no changes can be observed in terms of myofiber cross-sectional area and muscle strength in both sexes. However, divergent regulation has been reported in the same study at the molecular levels, suggesting that male mice harboring miR-21 deletion in osteocytes are more prone to activate muscle protein breakdown than females [16]. Another observation showed that the bone-forming activity of cultured osteoblasts is reduced by the exposure to miR-214 containing exosomes released by osteoclasts [17]. Consistently, the increased osteoclast expression of miR-214-3p was associated with high circulating levels of exosomes containing this microRNA and with reduced bone repair in both ovariectomized mice and aged women [18]. High circulating levels of osteoclast-derived miR-214 containing exosomes were also reported in patients with osteoporosis [18]. Experimental data showed a markedly different microRNA content between EVs released by MLO-Y4 osteocyte-cells compared to those produced by ST2 cells, a bone marrow-derived stromal cell line. Interestingly, 12 of those differentially expressed microRNAs were also reduced in the circulation of osteoclast-depleted mice [19]. Exosomes can also be released by mineralizing osteoblasts; several microRNAs detected into those exosomes were shown to be endowed with osteogenic potential, being able to drive the differentiation of ST2 cells into the osteoblastic lineage [20]. EVs derived from osteoblasts also contribute to both skeletal growth and bone mineral density; they accumulate in the unmineralized bone matrix by binding to extracellular matrix proteins and are identified as a peculiar subpopulation of EVs, defined as matrix vesicles (MtVs) [21]. These latter have been shown to contrast osteoclast activity, likely depending on the presence into the EV cargo of miR-125b, which targets the transcriptional repressor PR domain containing 1 (PRDM1), reducing its expression and resulting in increased levels of anti-osteoclastogenic factors [22]. Finally, miR-218 is another microRNA reported to be delivered by osteocyte-derived EVs and proposed to contribute to osteoblast differentiation by negatively regulating the expression of the osteokine sclerostin. Particularly relevant to the bone-muscle cross talk is the observation that the amount of EV-contained miR-218 can be reduced by exposing osteocyte cultures to the myokine myostatin [23].

#### Skeletal Muscle

Cancer-derived EVs containig miR-21 have been shown to contribute to myoblast apoptosis [24], partially explaining the impaired myogenesis occurring in both tumor-bearing animals and cancer hosts [25–27]. More recent data showed that EVs derived from the C26 colon carcinoma, a well-characterized cachexia-inducing murine tumor, negatively act on mitochondrial function and myogenesis in C2C12 myotube and myoblast cultures, respectively. The inhibition of myogenesis is likely due to miR-148a-3p and miR-181a-5p contained in the C26-derived EVs [28]. On the other side, the pharmacologic inhibition of EV release by C26 tumors was able to protect the host mice from cachexia. Such EVs were shown to contain high levels of miR-195a-5p and miR-125b-1-3p, which were supposed to play a causal role in cancer-induced muscle wasting. Consistently, the overexpression of those microRNAs in C2C12 cultures resulted in myotube reduction in size, an effect that was associated with the ability of miR-195a-5p and miR-125b-1-3p to downregulate Bcl_2_ expression, thus pushing the cell turnover balance towards apoptosis [29]. Similarly, miR-181a-3p encapsulated in EVs produced by oral squamous carcinoma cells was proposed to modulate Grp78 expression, leading to activation of the endoplasmic reticulum stress pathway, eventually resulting in C2C12 myotube thinning and in myoblast cell death [30]. Another study proposed that EV-contained miR-122 plays a role in breast cancer-associated muscle wasting by reducing the expression of O-linked N-acetylglucosamine transferase, a rate-limiting enzyme regulating the abundance of the ryanodine receptor. As a consequence of high miR-122 levels, the ryanodine receptor amount raises, resulting in increased cytosolic Ca^2+^ and calpain activation [31]. This latter is long known to operate a partial proteolysis of myofibrillar proteins, which become available for degradation by the ubiquitin-proteasome-dependent system, eventually leading to muscle protein wasting [32].

### Proteins

#### Bone

EVs containing RANK or RANKL, released by osteoclasts and osteoblasts/osteocytes, respectively, have been shown to regulate bone turnover. Indeed, osteoclasts and osteoblasts are generally non-contiguous and EV release takes care of conveying the reciprocal signals [33]. Indeed, RANKL-containing EVs have been shown to be produced by osteoblasts exposed to parathyroid hormone and to promote osteoclast survival [34]. The trafficking of RANKL containing EVs also occurs in zebrafish, supporting the relevance of such a mechanism through the evolution [35]. Exosomes released by M2 macrohpages (endowed with anti-inflammatory properties) were shown to contain colony stimulating factor (CSF)2, which appears able to inhibit osteoclast differentiation by inhibiting the TNFα-dependent signaling pathway [36]. A comparable inhibitory activty on osteoclastogenesis and bone resorption was displayed by EVs released by pericytes, in view of their ability to inhibit the transcription factor NF-κB by delivering TRAF3 (tumor necrosis factor receptor-associated factor-3; [37]). Finally, osteocyte release of EVs containing bone regulatory proteins, such as RANKL, osteoprotegerin, and sclerostin, is regulated by mechanotransduction [38].

#### Skeletal Muscle

In addition to microRNAs, proteins are among the molecules most present in EVs, either inside or on their surface. Several lines of evidence propose that at least some of these proteins can be relevant to muscle homeostasis. Some years ago, Hsp70 and Hsp90 bound to C26 tumor-derived EVs were reported to trigger muscle wasting with a mechanism involving the activation of a toll-like receptor (TLR) 4-p38 MAPK-intracellular protein breakdown axis [39, 40]. The existence of a relation between TLR4 and EV-associated proteins is confirmed by a different study showing the activation of TLR4-dependent signaling by HMGB1 contained into CT26-derived EVs. Consistently, HMGB1-ablated CT26 cells revealed unable to induce cachexia in the host mice and similar results were otained treating the animals with a HMGB1 inhibitor [41]. Another interesting finding was reported in a study investigating cachexia induced by ionizing radiation, the gold standard treatment for glioblastoma. The authors showed that after radiation exposure, glioblastoma cells release high amount of EVs containing plasminogen activator inhibitor-1 (PAI-1; [42]). Once delivered to the skeletal muscle, PAI-1 activates the transcription factor STAT3, which role in the onset and progression of muscle wasting is well known [43]. EVs released by the C26 cells have also been shown to contain growth and differentiation factor (GDF)15, which reduced the Bcl_2_/Bax ratio and increased caspase 3 cleavage in C2C12 cultures, resulting in myoblast apoptosis. Consistently, such an apoptosis-inducing pattern was abrogated when C2C12 cultures were exposed to exosomes derived from C26 cells rendered unable to express GDF15 [44].

## EVs as a Tool to Support Musculoskeletal Health

Physiologically, both muscle and bone are able to repair an injury by activating a regenerative program, which can be impaired in terms of triggering or progression by aging or by pathological states such as osteoporosis, sarcopenia, diabetes, and cancer. In this regard, strategies aimed at maintaining or restoring such a regenerative potential are currently under investigation. Particularly relevant in this regard is the possibility to take advantage of approaches based on MSCs. Along this line, especially for bone-related issues, some stem cell-based products recently reached the market and several clinical trials are actually ungoing [45]. The rationale underlying the use of MSCs is that, once implanted, they are able to engraft in the damaged bone/muscle and differentiate to reach the specific functional competence, eventually restoring tissue physiology. However, while on one side scaling up the availability of MSCs is not an easy task, on the other side, their therapeutic mechanism has recently been questioned, suggesting that MSC beneficial effect is mainly paracrine, due to the release in the microenvironment of biologically active factors, in particular those encapsulated into EVs. Indeed, these latter have been shown to be endowed with the ability to exert angiogenic and, depending on the site of damage, osteogenic or myogenic activities. In addition, the following features enforce the use of EVs rather then MSC infusion: (1) EVs easily reach the target organ, significantly reducing the risk of thrombi in the tissue capillary bed due to cell entrapment; (2) EVs display a low immunogenic and oncogenic ability compared to whole cells; (3) EVs can be functionalized to deliver mRNAs, non-coding RNAs, drugs, cytokines, and/or growth factors. Such properties render MSC-derived EVs a therapeutic tool more suitable than MSC transplantation.

Many recent studies appeared in the literature, which investigate the effectiveness of EVs in several experimental models of skeletal (fracture, osteoporosis in ovariectomized mice, bone loss induced by radiation) or muscle (aging, BaCl_2_, or cardiotoxin local injection) injury, showing improved tissue healing [46–48].

### Bone

The physiological recovery from bone fracture was markedly impaired in CD9^-/-^ mice, which release less EVs than wild-type animals, while improved when these animals were administered the exosome-containing conditioned medium of MSC cultures [49]. Another study showed that exosomes derived from MSCs preconditioned by hypoxia, administered to animals in which a bone fracture was induced, were able to repair bone damage more efficiently that exosomes derived from MSCs maintained in normoxia. Such an effect was associated with stimulation of angiogenesis and cell proliferation, and was mainly ascribed to the ability of the hypoxia-associated HIF-1α transcription factor to increase the exosome content in miR-126 [50]. Exosomes isolated from the culture medium of an adipocytic cell line (HS-5) were reported to contain nicotinamide phosphoribosyltransferase (NAMPT), a rate-limitying enzyme in the synthesis of NAD^+^ in mammals. When administered to rats in which intervetebral disc disease was induced by needle puncture, those exosomes were able to deliver their cargo to both nucleus pulposus cells and endplate cells in the intervertebral disc, resulting in rejuvenation of both cell populations by increasing intracellular NAD^+^ levels [51]. Osteoarthritis is one of the most relevant muscoloskeletal diseases. Along this line, treatments able to stimulate cartilage regeneration should be pursued. In the last few years, the possibility to use EVs is gaining a growing consensus. As an example, EVs released by bone marrow MSCs exposed to TGFβ3, shown to contain the chondrogenic microRNAs miR-455, were reported to improve osteoarthritis symptoms and cartilage replacement by activating the SOX11/FOXO signal transduction pathway [52]. These EVs also exert immunosuppressive and anti-inflammatory activities, resulting in a microenvironment that favors tissue regeneration [53]. Osteoblast-derived, miR-125b containing MtVs are endowed with anti-osteoclastogenic activity. Indeed, they proved able to counteract trabecular bone loss in different experimental models of osteoporosis [22]. Similarly, EVs derived from M2 macrophages have been reported to improve bone loss in female mice with ovariectomy-induced osteoporosis by delivering glutamate to osteoclasts, increasing the production of α-ketoglutarate. This latter interferes with an epigenetic program involving the gene Jmjd3, ultimately pushing the osteoclast/M2 macrophage balance towards macrophages [54]. Recent observations also point to organoid-derived EVs, apparently better than “classical” EVs in terms of amount recovered and effectiveness, as tools potentially useful to treat diseases affecting the bone [55]. Finally, exosomes derived from bovine milk have been reported to stimulate osteoblast proliferation and differentiation, and to inhibit osteoclast differentiation [56]. Similar observations were obtained investigating the effect of orally administered colostrum-derived exosomes to mice in which osteoporosis was induced by glucocorticoid treatment or oveariectomy, showing an improvement of bone mineral density associated with restored physiological microbiota [57, 58].

### Skeletal Muscle

Exosomes released by MSCs resident in the adipose tissue appeared effective in repairing surgically induced hind limb muscle damage, by stimulating satellite cell proliferation and differentiation [59]. EVs isolated by adipose tissue-MSCs maintained in hypoxia were able to improve cardiotoxin-induced muscle regeneration by increasing the EV content of miR-223, miR-146b, miR-126, and miR-199a [60]. Using the same experimental model, EVs obtained from adipose tissue-MSCs were reported to increase capillary density, likely due to their high content of vascular endothelial growth factor (VEGF)A and platelet and endothelial cell adhesion molecule (PECAM; [60]). Similar data were obtained using EVs released by MSCs of bone marrow origin [61]. Along the same line, exosomes released by cardiac progenitor cells proved effective in increasing myofiber number and in reducing inflammation and fibrosis in the skeletal muscle of *mdx* mice, an experimental model of Duchenne muscle dystrophy, also restoring, transiently at least, the expression of full-length dystrophin. Such a pattern was shown to be mainly associated with exosome-contained microRNAs [62, 63]. Conversely, inhibition of miR-21 in pancreatic cancer-derived EVs was reported to restore the myogenic capacity in primary myoblast cultures exposed to pro-inflammatory cytokines [24]. Human MSC-derived exosomes systemically administered to diabetic (*db/db*) mice and to animals rendered obese by high fat diet feeding resulted in increased muscle mass and function with a mechanism involving the stimulation of autophagy through an AMPK/ULK1 axis [64]. Muscle wasting induced by chemotherapy in healthy mice was reversed by miR-145-5p contained into EVs released by lymph node-derived MSCs, likely by interfering with the Activin A-dependent signaling pathway [65]. Similar observations were reported by a study showing that EVs of bone marrow MSC derivation contain miR-486-5p and can contrast dexamethasone-induced muscle wasting by inhibiting the activation of FoxO1, a transcription factor recognized to be crucially involved in the regulation of proteostasis. Consistently, such a protective action is antagonized by transfection with a miR-486-5p inhibitor [66]. The ability to exert an anabolic/anticatabolic action on the skeletal muscle was also demonstrated in bovine milk-derived exosomes. Indeed, in C2C12 myocyte cultures, they were reported to increased protein synthesis and myotube size, an effect that was associated with the presence of high levels of miR-149-3p and miR-2881 [67]. Differently to what reported in the bone, however, at present, such an anabolism-inducing pattern was not confirmed in the whole animal [56]. Finally, EVs isolated from the plasma of young mice revelaed able to rejuvenate aged satellite cells and to restore the muscle regenerative capacity. The underlying mechanism likely reflects the ability of young-derived EVs to improve the bioenergetic profile in the muscle by preserving mitochondrial function. This is associated with high mRNA levels of α-Klotho, a factor which positively modulates mitochondrial bioenergetics and muscle health. Indeed, when α-Klotho mRNA is lacking, the beneficial effect of young-derived EVs is lost [68].

## Conclusions

The idea of molecules transferred from one cell to another exists from the dawns of biology, being firstly suggested by Charles Darwin, who proposed a primordial hypothesis of inheritance mediated by particles defined as “*gemmules*” [69]. The discovery of EVs decades ago demonstrated that Darwin’s intuition was basically correct, although not in terms of inheritance. The present review highlights the relevance of EVs to the maintenance of the musculoskeletal homeostasis as well as to the muscle-bone cross-talk, eventually resulting in modulations of tissue microenvironment that impinge on cell death, proliferation, differentiation, and metabolism. In addition, insights into the possibility to use EVs as therapeutic tools is proposed. In this regard, several clinical trials are ongoing to test the feasibility of EVs for the treatment of musculoskeletal diseases and the preliminary data are encouraging [45]. However, several issues need to be further investigated to refine the therapeutic potential of EVs, such as better understanding the mechanisms by which specific cargoes are sequestered, directed, and delivered to target cells, to eventually exert a biological activity. Last, but not the least, increasing EV ability to escape degradation, to reach a specific tissue, and to deliver their cargo to the desired sites would markedly improve their translation to the clinical practice.

## Data Availability

No datasets were generated or analysed during the current study.
